# Supplementary material for: CHST7 Methylation Status Related to the Proliferation and Differentiation of Pituitary Adenomas
Source: Cells. 2022 Aug 4;11(15):2400. doi: 10.3390/cells11152400 (PMC9368070; doi:10.3390/cells11152400)

Figure S1. Correlation analysis of CHST7 and POU1F1 (A), SSTR2 (B) and DLL3 (C).

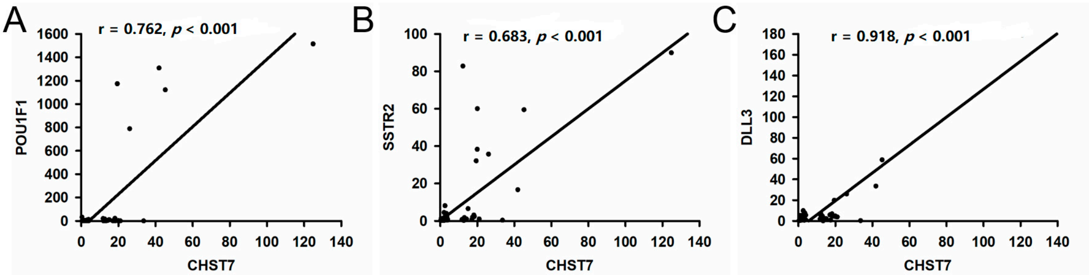

Figure S2. Correlatin analysis of CHSTY and mitochondrion-related genes. LAMP1 (A), OPA1 (B) and TOMM20 (C)

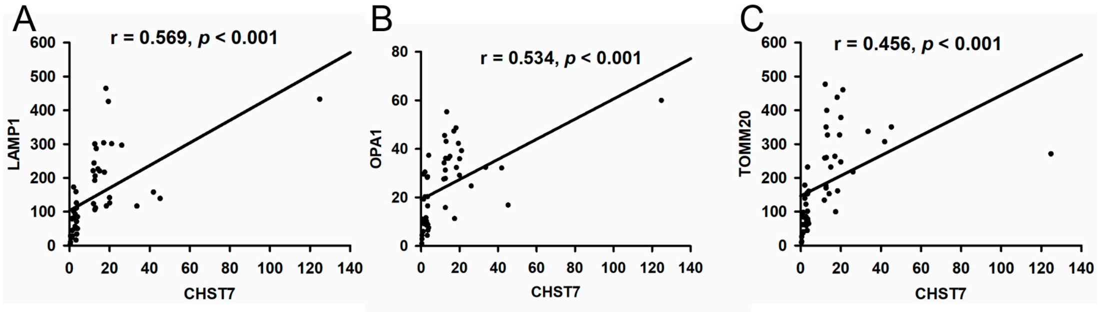

Figure S3. IHC pictures of transcription factors. Bar = 100 μm

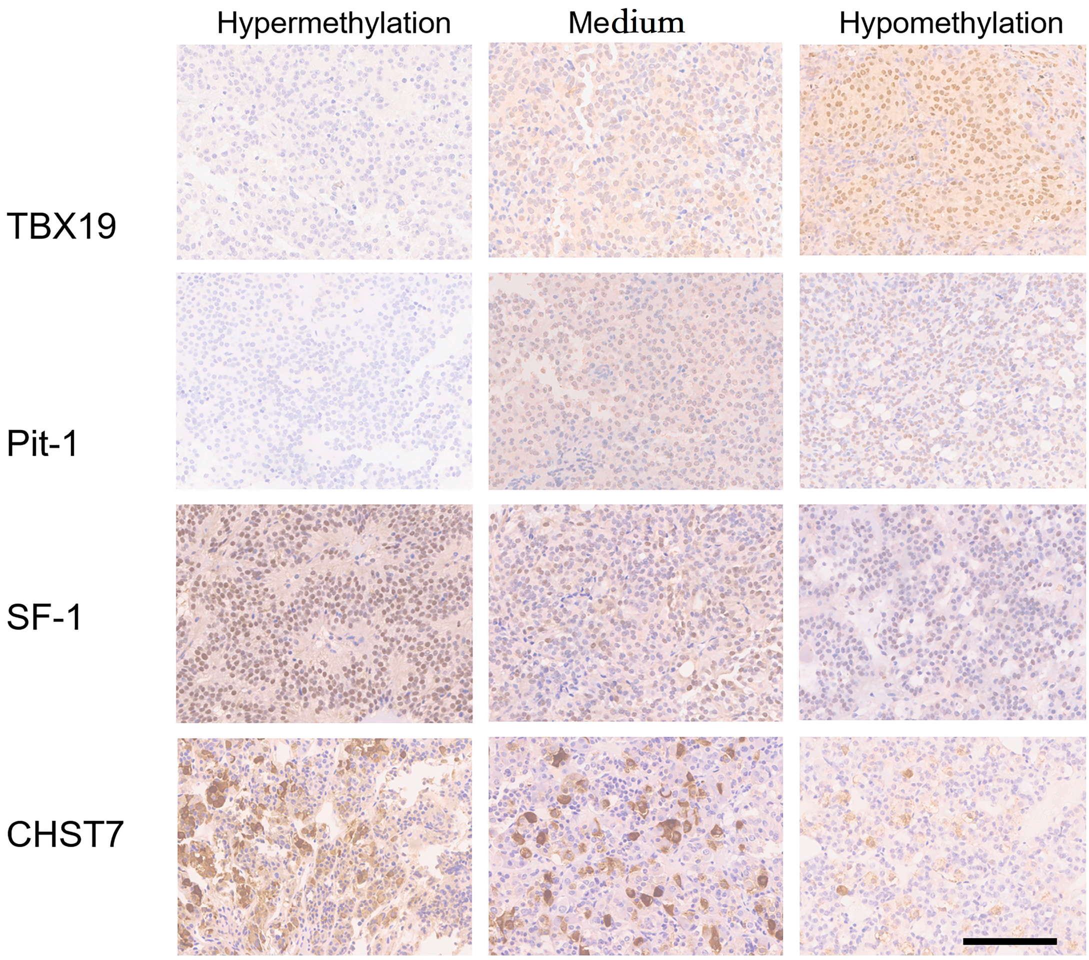

Figure S4. IHC pictures of receptors. Bar = 100 μm

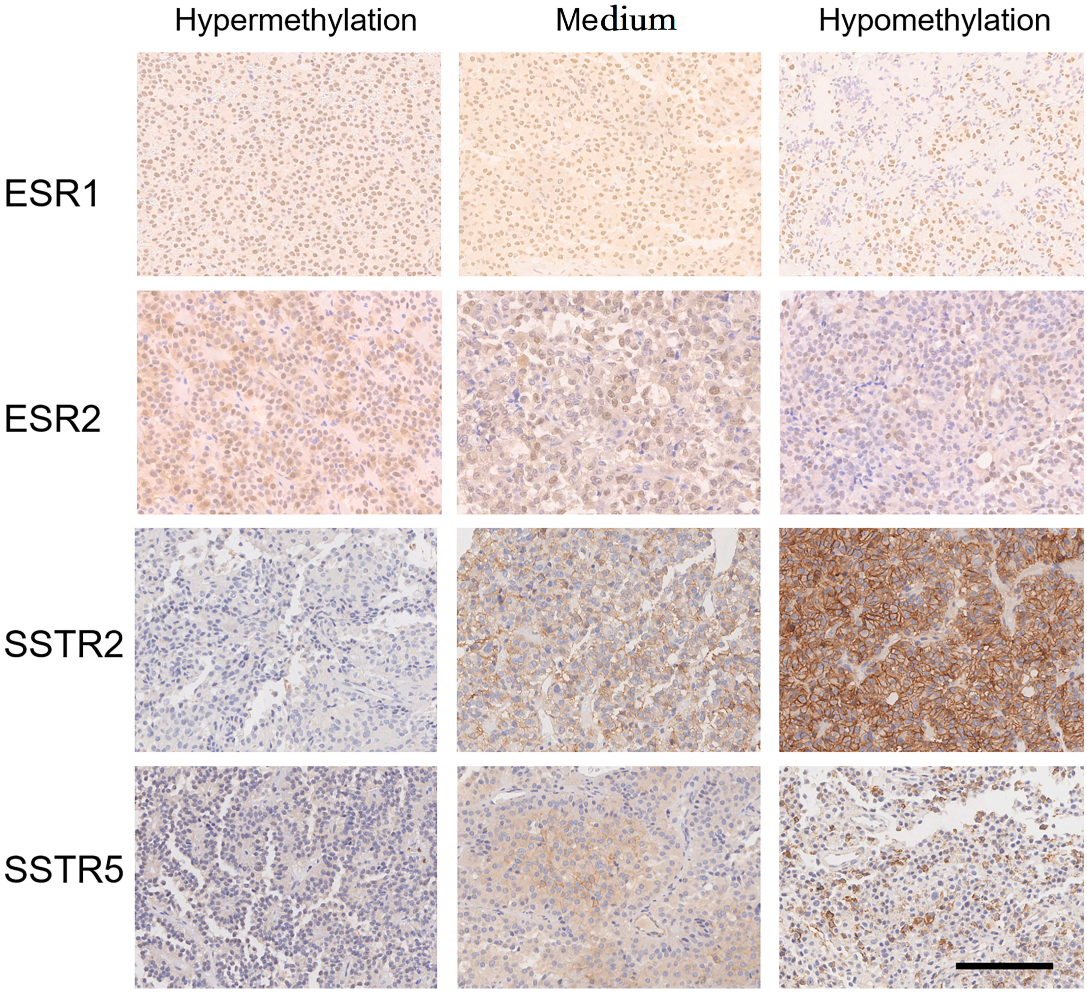

Supplement: Supplementary file 1 [file cells-11-02400-s001.zip › cells-1714604-supplementary.pdf]
